# Veratrum Viride in Puerperal Eclampsia

**Published:** 1879-06

**Authors:** 


### VERATRUM VIRIDE IN PUERPERAL ECLAMPSIA.

On the night of November 13th, 1878, was hurriedly called to see Mrs. A., living about three miles from town. The messenger could give no information as to the nature of the case, beyond the customary statement that she was "bad off."

On arrival found the patient, a young married woman in her first pregnancy, in the midst of a frightful convulsion. As soon as possible made a digital examination, to see whether she was in labor, and if so, how far advanced; found the os closed, and no sign of uterine contractions.

The woman was young and plethoric, and in all respects a good case for bleeding, with which I proposed to commence treatment; but my lancet case, which had not been out of my pocket in years, was now missing—and on examining my medicine chest my mortification was complete on finding I had neither chloroform nor chloral among its contents.

While I was internally debating whether I would return to town and provide myself with the necessary remedies, it occurred to me to try the effects of veratrum viride, of which I had a vial of Norwood's tincture.

The patient's pulse at that time was 156 per minute, the face congested and livid, and the breathing stertorous.

It is unnecessary to add that she was unconscious.

At that moment she had another convulsion, so terrible that I feared she would never come out of it alive.

Oh! how I wished for a lancet. However, before the fit was entirely over, I threw ten drops of Norwood's tincture into the arm at the insertion of the deltoid, and waited results. I did not have long to wait.

In ten minutes after insertion the pulse fell to 140; in five minutes more to 120; in ten more to 90, at which time a profuse sweat broke out over the whole body, the face became pale and the breathing less rapid; but the effects did not stop there. The pulse continued to fall rapidly until it got to 40, with intense sick stomach and vomiting of glairy mucus. The surface was cold and clammy, and the whole system completely relaxed. In a word, the tonic effects of the veratrum were present in an aggravated form. The situation began to be serious. The patient was still unconscious, the attendants supporting the head over the edge of the bed to assist in the effort at vomiting. It was impossible to give anything by the mouth, and the hypodermic administration of morphine seemed to be imperatively called for. Half a grain of sulph. morph. was inserted in the other arm, which in fifteen minutes quieted the retching; but the pulse still remained at 40, and the same profuse cold perspiration still bathed the surface of the body. There were no alcoholic stimulants in the house, and while a messenger was dispatched after some I concluded to try the hypodermic use of aromatic spirits of ammonia.

Half a drachm without dilution was injected into each arm, and before the messenger returned reaction set in. It was aided by hot bricks to the feet, spine and pit of the stomach.

I remained with the patient all night; she never had another symptom of convulsion, and became partly conscious in the morning, when her bowels were moved by an enema of salt and water, and she was left in a comfortable condition.

She was visited again in the evening, and was fully conscious. Some urine, which had been saved by me, was now examined and found to contain 60 per cent. of albumen. She had also considerable œdema of the feet and legs, and the face was puffy; but whether the result of albuminuria of the convulsions of the previous night, is not clear to me.

She was immediately placed on a diet of skimmed milk, of which the family had an abundance, and directed to take half ounce of bitartrate of potass. every alternate night until her confinement, which took place ten days afterwards. In five days there was only a slight appearance of albumen; but she had no convulsions, and passed safely through a severe and prolonged labor.

This case is reported because I think it proves the undoubted value of veratrum viride in eclampsia, and, according to a report of a police surgeon, published in the New York *Medical Record*, in the latter part of last year, it was of very great value combined with sulph. morphia in controlling epileptic convulsions.

If I were compelled to use it again in a similar case, I would not give more than five drops hypodermically, and repeat again if necessary; but although it might not act so powerfully in other cases as in this, it is well enough to be cautious in dealing with a powerful drug. In the May number, 1871, of the *American Journal of Obstetrics,* is a valuable article by Dr. Herbert Fearn, of Brooklyn, "on veratrum viride as a substitute for blood-letting in puerperal convulsions," which reports a large number of cases, all showing its great power in controlling eclampsia; but in the cases given it was administered per orem.

That we have, then, in veratrum viride another powerful weapon with which to combat this terrible disease, is clear to my mind; and to call the attention of the profession to it shall, I trust, be a valid excuse for reporting this case.—*N. C. Med. Journal.*

